# Cell Cycle Regulation by the PRMT6 Arginine Methyltransferase through Repression of Cyclin-Dependent Kinase Inhibitors

**DOI:** 10.1371/journal.pone.0041446

**Published:** 2012-08-20

**Authors:** Markus A. Kleinschmidt, Petra de Graaf, Hetty A. A. M. van Teeffelen, H. Th. Marc Timmers

**Affiliations:** Department of Molecular Cancer Research and Netherlands Proteomics Center, University Medical Center Utrecht, Utrecht, The Netherlands; Ludwig-Maximilians-Universität München, Germany

## Abstract

PRMT6 belongs to the family of Protein Arginine Methyltransferase (PRMT) enzymes that catalyze the methylation of guanidino nitrogens of arginine residues. PRMT6 has been shown to modify the tail of histone H3, but the *in vivo* function of PRMT6 is largely unknown. Here, we show that PRMT6 regulates cell cycle progression. Knockdown of PRMT6 expression in the human osteosarcoma cell line U2OS results in an accumulation of cells at the G2 checkpoint. Loss of PRMT6 coincides with upregulation of p21 and p27, two members of the CIP/KIP family of cyclin-dependent kinase (CDK) inhibitors. Gene expression and promoter analysis show that p21 and p27 are direct targets of PRMT6, which involves methylation of arginine-2 of histone H3. Our findings imply arginine methylation of histones by PRMT6 in cell cycle regulation.

## Introduction

Cell cycle progression is balanced by the activation and inhibition of cyclin-dependent kinases (CDKs). A variety of mechanisms have been identified that interfere with CDK activities, amongst them binding of INK4 and CIP/KIP inhibitors. Whereas INK4 proteins specifically impede G1/S transition by binding to CDK4 and CDK6, inhibitors of the CIP/KIP family are capable of interfering with the activity of a variety of CDKs [1].

The cell cycle regulator p21 (official gene symbol CDKN1A) becomes activated within several stress response pathways contributing to cellular fate decisions like cell cycle arrest, differentiation, senescence or apoptosis [2]. p21 exerts its function predominantly through inhibition of CDKs and of DNA synthesis. Despite its main characterization as a mediator of p53-dependent tumor suppressor activity several observations suggest an oncogenic potential of p21, presumably through its anti-apoptotic function and its ability to promote the assembly of cyclinD-CDK4 and -CDK6 complexes. p21 is one of three members of the CIP/KIP family of CDK inhibitors. The two other members, p27 (CDKN1B) and p57 (CDKN1C), also comprise an N-terminal CDK-inhibitory domain and have been implicated into cell cycle regulation [3]. As the family of CIP/KIP proteins inhibits all cyclin-CDK complexes, they are not specific for a particular phase in the cell cycle [4].

PRMT6 belongs to the family of Protein Arginine Methyltransferases (PRMTs), which are enzymes that catalyze the methylation of guanidino nitrogens of arginine residues. PRMT6 asymmetrically dimethylates arginine-2 of histone H3 (H3R2me2a) *in vivo* [5], [6], [7]. H3R2me2a correlates with inactive promoters in human cell lines and in the budding yeast, indicating a conserved function of this modification [5], [6], [8], [9]. PRMT6-mediated dimethylation of H3R2 prevents MLL/SET lysine methyltransferase complexes from binding to H3 [5], [6], [8], [9]. Hence, PRMT6 action impedes H3K4 trimethylation (H3K4me3), which is an active mark of transcription [10]. However, to date only a few direct transcriptional targets of PRMT6 have been described, amongst them the HoxA2 and TSP-12 genes [6], [11]. In mouse embryonic stem (ES) cells PRMT6 regulates pluripotency via direct binding to the promoter regions of *Oct4* and *Nanog* [12]. The process of PRMT6-mediated dimethylation of H3R2 needs to be tightly controlled in ES cells, since both knockdown and overexpression of PRMT6 induces differentiation. Recently, histone H2AR29 was described as a novel PRMT6 substrate [13]. Besides a role in transcriptional repression, PRMT6 regulates other cellular processes by methylating non-histone substrates. For example, PRMT6 methylates DNA polymerase beta to regulate base excision repair [14]. Furthermore, PRMT6 impairs HIV replication by methylating the viral proteins Tat and NC [15], [16].

Here we investigate the involvement of PRMT6 function in cell cycle regulation for several reasons. First, several PRMTs, namely PRMT1, CARM1/PRMT4 and PRMT5, have been implicated in cell proliferation and in the balance between pluripotency and differentiation [12], [17], [18], [19], [20], [21], [22], [23,]. Secondly, treatment with the general methyltransferase inhibitor AdOx results in activation of p21 in HUVEC cells [24] and in G2/M arrest in HeLa cells [25]. Thirdly, PRMT6 levels decline during replicative senescence [26]. Our results show that p21 and to a lesser extent p27, members of the CIP/KIP family of CDK inhibitors, are transcriptional targets of PRMT6. Knockdown of PRMT6 results in upregulation of both inhibitors, whereas expression of p57, the third member of the CIP/KIP family, remained unaltered. Overexpression of PRMT6 leads to an increase of H3R2 dimethylation at the transcriptional start sites of p21 and p27 promoter in U2OS cells, suggesting that both cell cycle inhibitor genes are direct targets of PRMT6. Loss of PRMT6 results in a delay of cell cycle progression. We observed an accumulation of cells with 4n chromosome content under PRMT6 knockdown conditions. Further analysis revealed that PRMT6 plays a role in G2 checkpoint regulation rather than mitosis.

## Results

### PRMT6 interferes with p21 and p27 expression

**Figure 1:**
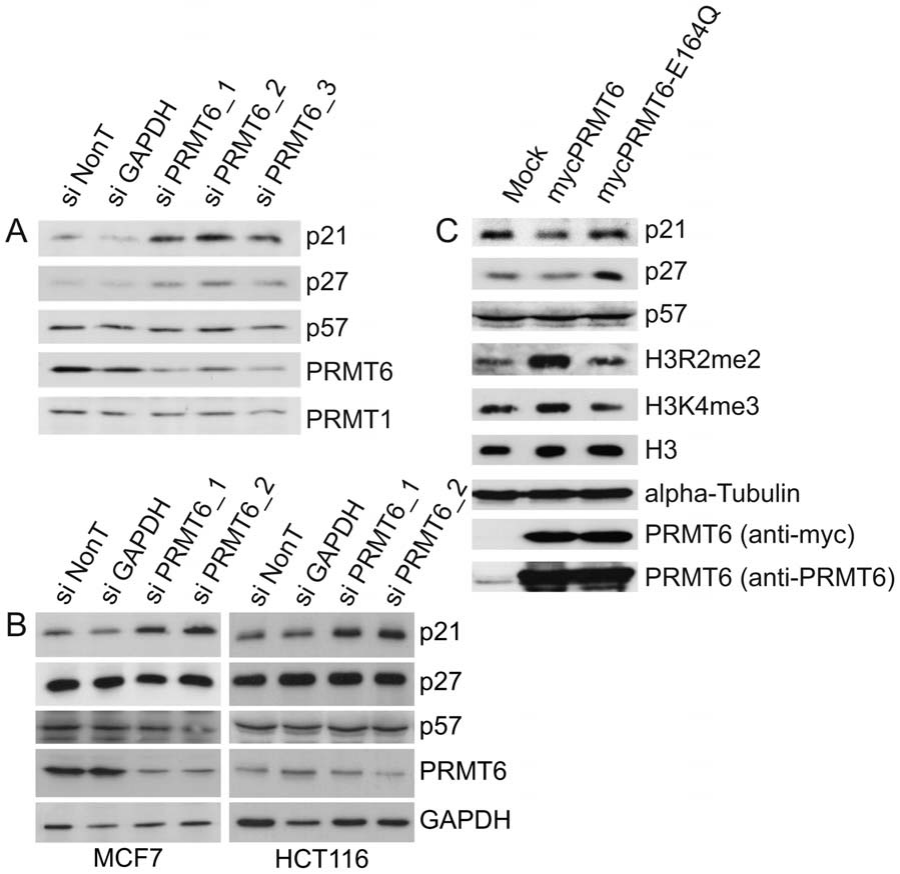
Knockdown of PRMT6 results in upregulation of p21. (A) U2OS cells were transfected with the indicated siRNAs. After 72 hours cells were harvested and immunoblot analysis was performed using antisera against PRMT6, PRMT1, p21,p27 and p57. (B) MCF7 (right panel) or HCT116 (left panel) cells were transfected with the indicated siRNAs. After 72 hours cells were harvested and immunoblot analysis was performed using antisera against PRMT6, p21, p27, p57 and GAPDH. (C) U2OS cells were transfected with expression constructs for pcDNA3-PRMT6 wild type and pcDNA3-PRMT6-E164Q or untreated (mock). After 48 hours cells were harvested and analysed with the indicated antisera.

The expression of PRMT6 and the p21 proteins are inversely related in an experimental system for cellular aging [26]. To address a functional relation between PRMT6 and p21 we reduced PRMT6 expression by siRNA mediated knockdown in the human osteosarcoma cell line U2OS and analyzed protein expression level of all three members of the KIP/CIP family, namely p21, p27 and p57. As shown in Figure 1A, transfection of three independent siRNAs directed against PRMT6 led to an efficient knockdown of the enzyme. PRMT1 served as a loading control, indicating the specificity of the knockdown procedure. Interestingly, p21 expression increased upon PRMT6 knockdown conditions but not upon transfection of our control siRNAs (NonTargeting and GAPDH). Notably, p27 was weakly upregulated under these conditions, whereas p57 level remained unaltered. Knockdown of PRMT6 in MCF7 cells also increased p21 expression (Fig. 1B). Despite only a small reduction in PRMT6 levels after siRNA-mediated knockdown in HCT116 cells, p21 expression is increased significantly (Fig. 1B). However, upregulation of p27 was not observed in both cell lines.

The effect of PRMT6 overexpression on CIP/KIP inhibitor expression was further investigated in the U2OS cell system. As expected, high PRMT6 level correlated with a global rise of the H3R2me2a mark (Fig. 1C). Analysis of the CIP/KIP inhibitor level revealed a detectable downregulation of p21 and p27 but not of p57. In order to confirm that these effects rely on the active enzyme the effects of a catalytically inactive mutant of PRMT6 (E164Q) were investigated. The glutamic acid at position 164 is a critical residue of the double E loop, a motif required for the enzymatic activity of PRMTs [27], [28]. Expression of PRMT6-E164Q did not alter H3R2me2a levels nor suppressed p21 or p27 expression (Fig. 1C). Interestingly, we noticed higher levels of p27 compared to mock condition, which may indicate a selective dominant-negative effect of the PRMT6 mutant. Taken together, these results indicate that PRMT6 acts to reduce the expression of p21.

### PRMT6 regulates p21 and p27 expression at the transcriptional level

**Figure 2:**
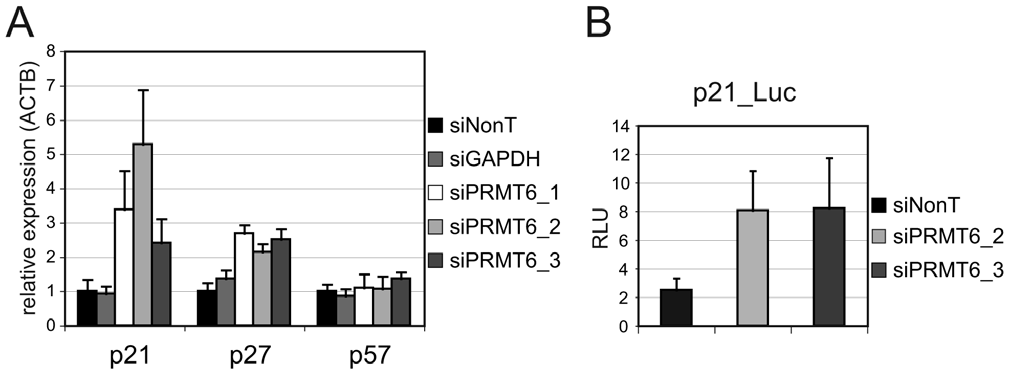
Transcriptional regulation of p21 and p27 by PRMT6. (A) U2OS cells were transfected with the indicated siRNAs and harvested 72 h later. RT-qPCR was performed with primers for p21, p27 and 57. Gene expression was normalized for beta-Actin. Error bars indicate the standard error of the mean. (B) Cells were transfected with p21-Luc reporter and the indicated siRNAs. Relative light units represent the mean value of a triplicate transfection.

PRMT6 has been described as a transcriptional repressor for the HoxA2 and TSP-1 genes [6], [11]. Therefore, we hypothesized a similar mechanism for p21 and p27 regulation. To address this, quantitative PCR was performed to analyze mRNA expression of these CDK inhibitors after PRMT6 knockdown. As expected, mRNA levels correlated with protein expression levels (Fig. 2A and 1A). PRMT6 knockdown increased the amount of both p21 and p27 transcripts with the strongest effect on p21. Next we tested whether PRMT6 acts at the transcriptional level as this protein has also been involved in post-transcriptional processes [29]. To this end transfection of a Firefly-Luciferase reporter gene under the control of the human p21 promoter was combined with PRMT6 siRNAs. Figure 2B shows that knockdown of PRMT6 expression led to a three-fold induction of the reporter gene activity. Taken together, these experiments indicate that PRMT6 regulates p21 expression at the transcriptional level.

### PRMT6 expression increases H3R2 methylation of the p21 and p27 promoters

**Figure 3:**
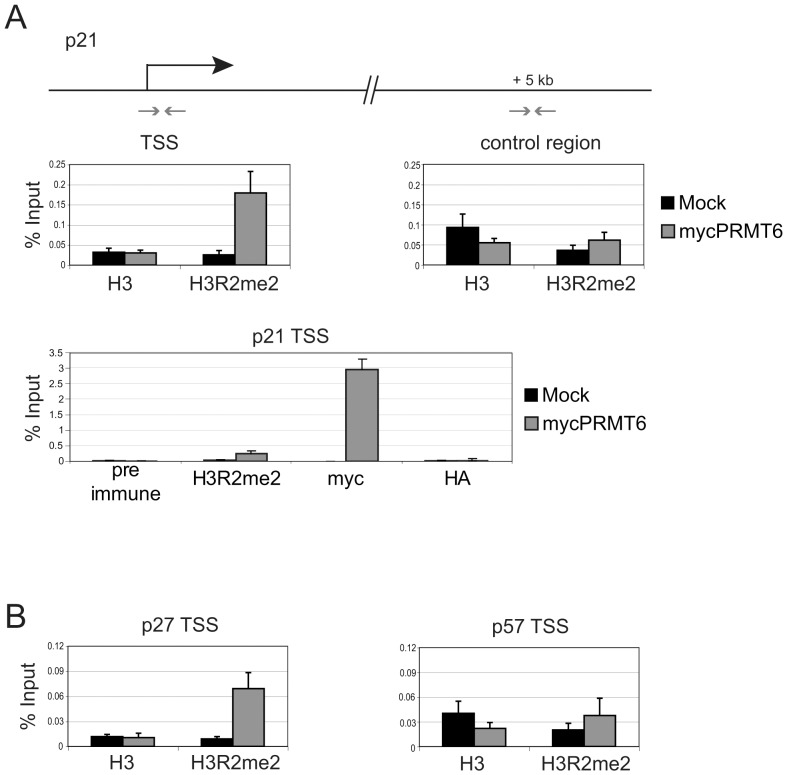
PRMT6 regulates p21 and p27 on the promoter level. (A) ChIP analysis was done 46 h upon transient expression of mycPRMT6 using primers for the transcriptional start site (TSS) and a control region of the p21 promoter. (B) ChIP analysis as in A, using promoter specific primers for the TSS of p27 and p57.

PRMT6 has been shown to methylate R2 of histone H3 and this asymmetrical dimethylation of H3R2 correlates with promoter repression [5]. As overexpression of PRMT6 leads to a global rise of the H3R2me2a mark in U2OS cells (Fig. 1C), we examined whether H3R2me2a levels at the p21 promoter are also affected by PRMT6. To this end, primers mapping to the transcriptional start site (TSS) of the p21 promoter were used for qPCR analysis after chromatin immunoprecipitation (ChIP). ChIPs were performed using antibodies for myc to detect transiently expressed PRMT6, H3R2me2a-modified and for total histone H3. As expected, PRMT6 is recruited to the TSS of p21 upon ectopic expression, which coincides with increased H3R2 dimethylation levels (Fig. 3A). As a control we performed qPCR analysis for a region 5 kb downstream of the TSS. Here, H3R2me2a levels were not enriched over H3. We determined the presence of H3R2me2a at the p27 and p57 promoters under the same conditions (Fig. 3B). Similar to p21, H3R2 dimethylation was enriched at the p27 promoter. However, there was no significant change of H3R2me2a at the TSS of the p57 promoter. These results indicate a direct role for PRMT6 in repression of p21 and p27 promoter activity.

### Accumulation of cells in G2 upon knockdown of PRMT6

**Figure 4:**
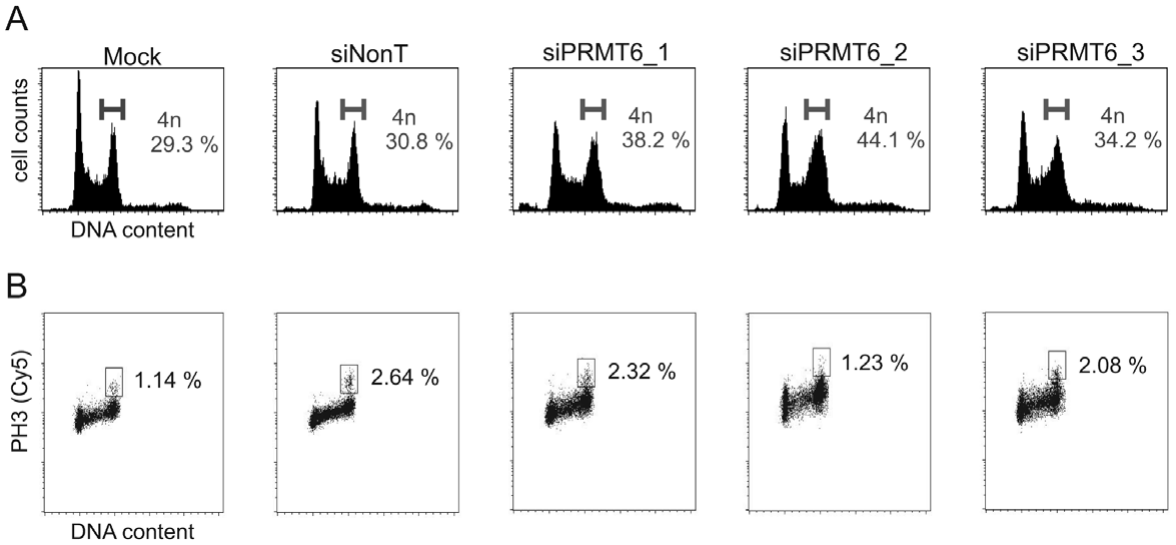
Knockdown of PRMT6 results in an accumulation of cells in G2. (A) U2OS cells were transfected with the indicated siRNAs and fixed 72 h later. For DNA content analysis, cells were stained with propidium iodide and analyzed by flow cytometry. Percentage of cells with a 4n DNA content is indicated. This is a representative example of three biological replicates. (B) Same as in A, this time cells were double stained using propidium iodide and Phospho-H3S10 antibody. Percentage of mitotic cells is indicated.

CIP/KIP family members impede cell cycle progression at a variety of checkpoints. As both p21 and p27 are expressed upon loss of PRMT6, we asked whether this results in cell proliferation defects. Three independent siRNAs targeting PRMT6 were transfected into the human osteosarcoma cell line U2OS. After 72 hours the cell cycle profile was analyzed by FACS (Fig. 4A and Table 1). Transfection of each PRMT6 siRNA resulted in an increase of the 4n chromosome content compared to mock or Non-Targeting siRNA transfected cells. Since 4n cell cycle checkpoints exist both in G2 and mitotic cells, we further investigated the cell cycle defects of siPRMT6-transfected cells. Phosphorylation of histone H3 serine 10 (H3S10) is a mitotic mark in eukaryotic cells. Staining and quantification of H3S10ph-positive cells allows distinction between G2 and mitotic arrest [30]. We investigated mitotic populations after PRMT6 knockdown by means of an H3S10ph antibody and flow cytometry and this was plotted against DNA content (Fig. 4B). Clearly, the number of H3S10ph positive cells did not increase under PRMT6 knockdown conditions. These results suggest, that loss of PRMT6 expression in U2OS cells leads to activation of a G2 checkpoint rather than a mitotic checkpoint.

**Table 1:** Cell cycle distribution of control and siPRMT6 transfected U2OS cells.

*Condition*	*G1*	*S*	*G2/M*	*>4n*
Mock	36.0	28.0	29.3	5.8
siNonT	35.0	25.5	30.8	7.8
siPRMT6_1	31.6	18.7	38.2	9.5
siPRMT6_2	28.9	15.9	44.1	10.4
siPRMT6_3	31.9	23.2	34.2	9.6

Conditions as described in the legend of Figure 4A. Missing percentage: apoptotic cells (<2n).

## Discussion

Here, we report a role for PRMT6 in cell cycle regulation and show that PRMT6 negatively regulates p21 protein expression in three human cell lines tested. Expression of PRMT6 leads to increased levels of H3R2me2a on the p21 and p27 promoters in U2OS cells, leading to reduced mRNA and protein expression of these genes. Furthermore, knockdown of PRMT6 and increased p21 and p27 levels coincide with an accumulation of cells in G2. This is in accordance with the finding that overexpression of p21 results in CDK1 inhibition and G2 arrest in U2OS cells [31].

Overexpression of PRMT6 leads to a weak but detectable downregulation of p21 and p27 expression. Possibly, PRMT6 action requires assistance by co-regulators to fully exert its repressive function. To date only a few interactors of PRMT6 have been described in the literature, the HIV proteins Tat and Ref, and the human DNA polymerase beta [14], [16], [32]. However, it has been shown before that PRMT6 regulates transcription through recruitment to target promoters [6]. Since PRMT6 lacks a DNA binding domain, we postulate the existence of additional binding partners of PRMT6 for efficient promoter targeting. At present such PRMT6-interacting proteins remain to be identified and this may involve transient interactions as PRMT6 has been shown to sediment as a monomer in glycerol gradients [33]. Moreover, it is not clear whether the cell cycle effects under PRMT6 knockdown conditions solely rely on the activation of p21 and p27.

PRMT6-mediated dimethylation of H3R2 prevents MLL/SET lysine methyltransferase complexes from binding to H3 and, hence, it impedes H3K4 trimethylation (H3K4me3) [5], [6]. In addition, H3R3me2a modification has been shown to inhibit H3K4me3-binding of effector molecules like TFIID, BPTF-NURF or ING2-Sin3A [10], [34]. In line with our results, recent publications suggest that menin, a member of the MLL complex, functions as a tumor suppressor through transcriptional activation of CDK inhibitors. Using knockout MEFs, menin has shown to be required for activation of the p21 gene in DNA damage response [35]. Moreover, MLL associates with and activates p27 and p18 promoters in a menin-dependent manner [36].

Interestingly, another arginine methyltransferase, CARM1/PRMT4, regulates the half-life of the p21 transcript through methylation of the mRNA binding protein HuD [17]. The authors suggest that methylated HuD maintains PC12 cells in a proliferative state by committing p21 mRNA to its decay system. Loss of CARM1/PRMT4 leads to increased p21 protein level, whereas p27 expression is not affected. Recently, CARM1/PRMT4 and PRMT6 were found to be overexpressed in a variety of human cancers compared to non-neoplastic tissues. PRMT6 knockdown reduced the proliferation of bladder and lung cancer cell lines, as a result of a block in cell cycle progression [37]. These and our findings that enzyme activities of CARM1/PRMT4 and PRMT6 are capable of regulating tumor suppressor activity and cellular proliferation raise questions of a common meaning for cellular metabolism.

Arginine Methyltransferase activity depends on the methyl-group donor, S-Adenosylmethionine (SAM). SAM is provided by the methionine cycle, which in turn requires uptake of the essential amino acid methionine and additional methyl-group donors. Demethylated metabolites of the methionine cycle rise to abnormal high levels in cancer cells [38]. These products, i.e. S-Adenosylhomocysteine (SAH) and Methylthioadenosine (MTA), are known inhibitors of S-Adenosylmethionine (SAM) dependent methyltransferases. With the identification of PRMT6 and CARM1 as inhibitors of p21 and p27 a novel link between the methylation cycle and tumor suppression is pointed out. To summarize, we present evidence that PRMT6 regulates the eukaryotic cell cycle at the G2 checkpoint. This involves transcriptional regulation of p21 and p27 via elevated levels of H3R2me2a at promoters through the enzymatic activity of PRMT6. Since p21 has been discussed as a promising target for cancer therapeutics, modulation of PRMT6 activity should be considered as a target of pharmaceutical drug design [2], [39].

## Materials and Methods

### Cell Culture & Transfections

Human osteosarcoma cell line U2OS (#HTB-96), breast adenocarcinoma cell line MCF-7 (#HTB-22) and colorectal carcinoma cell line HCT116 (#CCL-247) were obtained from the ATCC and cultured in Dulbecco's modified Eagle's medium (DMEM), supplemented with 10% fetal bovine serum, 2 mM L-glutamine, 100 U/ml penicillin and 100 µg/ml streptomycin. SiRNA oligonucleotide duplexes were purchased from Dharmacon and Eurogentec. PRMT6 targeting sequences (sense orientation) are as follows GAGCAAGACACGGACGUUU (siPRMT6_1), GCACCGGCAUUCUGAGCAU (siPRMT6_2), CGGAUACAGCGUGCUUAUUAU (siPRMT6_3). SiGENOME Non-Targeting siRNA Pools (D-001206-13) and siGENOME GAPDH control siRNA (D-001140-01) were purchased from Dharmacon. SiRNA duplexes were transfected with Oligofectamine at a final concentration of 80 nM for U2OS and 50 nM for HCT116 and MCF-7 cells according to manufacturer's advice. For transient overexpression 5 µg pcDNA-Mock, pcDNA-mycPRMT6 or pcDNA-mycPRMT6-E164Q, respectively, were transfected into U2OS in a 10 cm cell culture dish using Fugene 6 (Roche). PRMT6 expression constructs were generous gifts from Michael Hottiger.

### Antibodies

The following antibodies were used: H3 (Abcam, ab1791), H3R2me2a (Upstate, 07-585), alpha Tubulin (Sigma, T6793), GAPDH (Millipore, 6C5), myc-tag 9E10 (Roche), p21 (Santa Cruz), p27 (BD Pharmingen, 554069) and p57 (Santa Cruz, sc-1040). The PRMT6 rabbit antisera were a kind gift from Uta-Maria Bauer [40].

### Reporter gene assay

U2OS cells were plated in 24-well plates. At the next day, cells were transfected at a confluency of 50% with 0.2 µg of p21-Luc [41], 0.12 µg pCMV-Renilla and 40 pmol of indicated siRNA Oligos using Lipofectamine 2000 (Invitrogen). Medium was changed 48 h after transfection. 72 h upon transfection cells were lysed and analyzed for Luciferase activity by means of Promega's Dual Luciferase Reporter Assay System. Each transfection was performed in triplicates. Error bars present the standard deviation of the triplicates in a representative experiment.

### RNA isolation & RT-qPCR

Detailed information regarding reverse transcriptase (RT)-qPCR procedure has been described before [42]. Briefly, total RNA was purified by means of an RNeasy Mini Kit (Qiagen) according to the manufacturer's protocol. 0.5 µg of RNA were applied to reverse transcription using SuperScriptII (Invitrogen) and oligo(dT)17 primer. Quantitative PCR was carried out using the AmpliTaq Gold Kit (Applied Biosystems) and analyzed on a BioRad C1000 cycler. For gene expression analysis the following primers were used:

hACTB_fwd (AGAAAATCTGGCACCACACC),
hACTB_rev (AGAGGCGTACAGGGATAGCA),
h_p21_fwd (TCACTGTCTTGTACCCTTGTGC),
h_p21_rev (GGCGTTTGGAGTGGTAGAAA),
h_p27_fwd (TGACTTGCATGAAGAGAAGCA),
h_p27_rev (GCTGTCTCTGAAAGGGACATTAC),
h_p57_fwd (GAGCGAGCTAGCCAGCAG) and
h_p57_rev (GCGACAAGACGCTCCATC).

### ChIP assays

Chromatin immunoprecipitation was essentially done as described [43]. For quantitative PCR the following primers were used:

h_p21_TSS_fwd (TGCGTTCACAGGTGTTTCTG),
h_p21_TSS_rev (CACATCCCGACTCTCGTCAC),
h_p21_cr_fwd (AAGTGATTGTGATGGGCCTC),
h_p21_cr_rev (TGAACCCCACTCCCTCTCTA),
h_p27_TSS_fwd (ACTCGCCGTGTCAATCATTT),
h_p27_TSS_rev (AACACCCCGAAAAGACGAG),
h_p57_TSS_fwd (TCCAGCTCTCCAGCTTTTG) and
h_p57_TSS_rev (TCCAGTCTGTTTGTGCTTGTG).

### Flow cytometry

For DNA content analysis cells were harvested by trypsinization and resuspended in DMEM. Subsequently, cells were washed twice in ice-cold PBS and fixed in 80% ethanol at −20°C. For mitotic index analysis cells were incubated for 1 h at RT with Phospho-H3S10 antibody (Upstate) diluted 1∶500 in PBS/0.05% Tween/2% BSA. Afterwards, cells were washed twice with PBS/0.05% Tween and incubated for 1 h at RT with secondary Goat anti-Rabbit Cy5 antibody (Jackson Laboratories). DNA was stained with 69 µM propidium iodide in 38 mM sodium citrate and 100 µg/ml RNase A for 30 min at 37°C. Samples were analyzed in a Becton Dickinson FACS Calibur.
